# A Histological and Clinical Study of MatriDerm® Use in Burn Reconstruction

**DOI:** 10.1093/jbcr/irad024

**Published:** 2023-03-22

**Authors:** Kathryn Dickson, Kwang Chear Lee, Abdulrazak Abdulsalam, Ezekwe Amirize, Hadyn K N Kankam, Britt ter Horst, Fay Gardiner, Amy Bamford, Rahul K Hejmadi, Naiem Moiemen

**Affiliations:** University Hospital Birmingham NHS Foundation Trust, Queen Elizabeth Hospital, Mindelsohn Way, Edgbaston, Birmingham B15 2WB, UK; University Hospital Birmingham NHS Foundation Trust, Queen Elizabeth Hospital, Mindelsohn Way, Edgbaston, Birmingham B15 2WB, UK; Scar Free Foundation Centre for Conflict Wound Research, University Hospital Birmingham NHS Foundation Trust, Queen Elizabeth Hospital, Mindelsohn Way, Edgbaston, Birmingham B15 2WB, UK; Institute of Inflammation and Aging, University of Birmingham, Birmingham B15 2TT, UK; University Hospital Birmingham NHS Foundation Trust, Queen Elizabeth Hospital, Mindelsohn Way, Edgbaston, Birmingham B15 2WB, UK; Scar Free Foundation Centre for Conflict Wound Research, University Hospital Birmingham NHS Foundation Trust, Queen Elizabeth Hospital, Mindelsohn Way, Edgbaston, Birmingham B15 2WB, UK; University Hospital Birmingham NHS Foundation Trust, Queen Elizabeth Hospital, Mindelsohn Way, Edgbaston, Birmingham B15 2WB, UK; Scar Free Foundation Centre for Conflict Wound Research, University Hospital Birmingham NHS Foundation Trust, Queen Elizabeth Hospital, Mindelsohn Way, Edgbaston, Birmingham B15 2WB, UK; University Hospital Birmingham NHS Foundation Trust, Queen Elizabeth Hospital, Mindelsohn Way, Edgbaston, Birmingham B15 2WB, UK; Institute of Inflammation and Aging, University of Birmingham, Birmingham B15 2TT, UK; University Hospital Birmingham NHS Foundation Trust, Queen Elizabeth Hospital, Mindelsohn Way, Edgbaston, Birmingham B15 2WB, UK; Scar Free Foundation Centre for Conflict Wound Research, University Hospital Birmingham NHS Foundation Trust, Queen Elizabeth Hospital, Mindelsohn Way, Edgbaston, Birmingham B15 2WB, UK; University Hospital Birmingham NHS Foundation Trust, Queen Elizabeth Hospital, Mindelsohn Way, Edgbaston, Birmingham B15 2WB, UK; Scar Free Foundation Centre for Conflict Wound Research, University Hospital Birmingham NHS Foundation Trust, Queen Elizabeth Hospital, Mindelsohn Way, Edgbaston, Birmingham B15 2WB, UK; University Hospital Birmingham NHS Foundation Trust, Queen Elizabeth Hospital, Mindelsohn Way, Edgbaston, Birmingham B15 2WB, UK; Scar Free Foundation Centre for Conflict Wound Research, University Hospital Birmingham NHS Foundation Trust, Queen Elizabeth Hospital, Mindelsohn Way, Edgbaston, Birmingham B15 2WB, UK; University Hospital Birmingham NHS Foundation Trust, Queen Elizabeth Hospital, Mindelsohn Way, Edgbaston, Birmingham B15 2WB, UK; University Hospital Birmingham NHS Foundation Trust, Queen Elizabeth Hospital, Mindelsohn Way, Edgbaston, Birmingham B15 2WB, UK; Scar Free Foundation Centre for Conflict Wound Research, University Hospital Birmingham NHS Foundation Trust, Queen Elizabeth Hospital, Mindelsohn Way, Edgbaston, Birmingham B15 2WB, UK; Institute of Inflammation and Aging, University of Birmingham, Birmingham B15 2TT, UK

## Abstract

Dermal substitutes are well established in the reconstructive ladder. MatriDerm® (Dr. Otto Suwelack Skin & Health Care AG, Billerbeck, Germany) is a single-layer dermal substitute composed of a bovine collagen (type I, III, and V) and elastin hydrolysate, that allows for immediate split-thickness skin grafting (SSG). The aim of this study was to histologically characterize the integration of MatriDerm® when used during burns surgery reconstruction. Eight subjects with nine burn scars and one acute burn wound underwent reconstruction with MatriDerm® and an immediate SSG. MatriDerm® integration and skin graft take were assessed with serial biopsies performed at weeks 1, 2, 3, and 4 and months 2, 3, 6, 9, and 12. Biopsies were assessed with standard special stains and immunohistochemistry, and representative slides were imaged with a transmission electron microscope. Patient satisfaction and clinical scar outcome were assessed with the Vancouver Scar Scale and a patient questionnaire. Histological analysis showed similar stages of wound healing as shown in other dermal templates but on a different timescale. There is early evidence of vascularization and an inflammatory infiltrate in the first 2 weeks. MatriDerm® is resorbed earlier than other dermal substitutes, with evidence of resorption at week 3, to be completely replaced by a neodermis at 2 months. The use of MatriDerm® in reconstruction with immediate skin grafting is supported histologically with early evidence of vascularization to support an epidermal autograft. Future histological studies may help further characterize the ideal dermal substitute.

Global mortality secondary to burn injury continues to decrease,^1^ making the attainment of satisfactory functional outcomes and acceptable aesthetic appearance now paramount. Dermal substitutes were originally developed as a solution to the challenge of limited donor sites by providing alternative wound cover, but have been increasingly used to address scar quality, with favorable functional and aesthetic outcomes.^2–6^

MatriDerm® (Dr. Otto Suwelack Skin & Health Care AG, Billerbeck, Germany) is a single-layer dermal substitute composed of bovine collagen (types I, III, and V) and elastin hydrolysate. The collagen–elastin template creates a porous matrix facilitating the immediate application of a skin graft onto the 1 mm thick sheet. Immediate application of skin graft over a dermal matrix is a new concept. The matrix can act as a barrier for vascularization and may lead to skin graft desiccation and failure. A delay in revascularisation of the skin graft due to the presence of MatriDerm®, was not observed in high-resolution episcopic microscopy, and it was suggested that nutritional support of the skin graft is provided through diffusion, preventing graft desiccation^.7^

MatriDerm® collagen is not cross-linked, unlike other dermal substitutes. Cross-linking of a dermal matrix results in a more stable structure that has increased resistance to biodegradation, which can be observed clinically with MatriDerm® having been found to absorb more quickly when compared to other dermal substitutes.^8^ However, increasing a dermal substitute’s rigidity through cross-linking may cause increased differentiation of fibroblasts to myofibroblasts, which could lead to increased wound contracture.^9^ A study examining the effect of cross-linking MatriDerm® using the same technique as that used to cross-link Integra® found that cross-linked MatriDerm® had reduced pore size and density, and delayed evidence of angiogenesis when compared to noncross-linked MatriDerm®. Biodegradability may have a relationship to angiogenesis, with vessel formation primarily observed in areas where the scaffold had biodegraded. Ultimately this did not affect graft take or wound contraction in an in vivo murine model.^10^ MatriDerm® matrix design has been shown to be crucial in influencing neovascularization, with a large pore size and relatively low density, allowing diffusion of nutrition and faster ingrowth of host fibroblasts and endothelial cells forming new vascular channels and neodermis.^11–13^

Other dermal substitutes such as bi-layered Integra® (Integra LifeSciences Corporation, Plainsboro, USA)^14–16^ and Pelnac (Gunze, Osaka, Japan),^17–20^ use a temporary silicon layer which protects the dermal substitute from fluid loss and infection whilst matrix vascularization and neodermis formation take place. The silicon layer is replaced with autologous split-thickness skin graft (SSG) when wound bed integration is complete, as a two-stage reconstruction procedure. More recently the development of NovoSorb™ Biodegradable Temporizing Matrix (Polynovo, Adelaide, Australia), also a bi-layer dermal substitute, has shown that animal products can be avoided, being a fully synthetic matrix composed of a biodegradable polyurethane foam.^21–24^

MatriDerm® provides a scaffold for the formation of a neodermis when applied to full-thickness wounds, in the context of acute burn excision, skin cancer, trauma, or scar resurfacing.^6,25,26^ In the acute burn setting, MatriDerm® can be prioritized for areas of cosmetic or functional importance.^6,27–29^ It has also been shown to have a role in areas in which skin grafting would not be effective, such as bone without periosteum and tendon without paratenon.^30^ The long-term clinical and histological benefits of MatriDerm® over skin grafting alone are yet to be established.^31,32^

We have previously examined the histological profile of Integra® and its ability to form a neodermis, providing evidence for the optimal timing of grafting in the use of Integra®.^33^ A similar study has not been conducted for MatriDerm®. The aim of this study was to characterize the histological changes of MatriDerm® during the various stages of its integration into the wound bed and to assess how this process differs from that of Integra®. Our current surgical protocol for use of MatriDerm® is also described.

## METHODS

Ethical approval was obtained from the Regional Ethics Committee (NRES Committee West Midlands-South Birmingham—REC reference 12/WM/0024) prior to commencement of the study.

### Patient Recruitment and Selection

Patients requiring reconstruction for acute burn injuries or burn scars were screened from inpatient multidisciplinary ward rounds and outpatient clinics. Patients with a total of ten reconstructed sites meeting the eligibility criteria (Table 1) were approached using patient information sheets to obtain informed consent. Baseline data including demographics, comorbidities, and Fitzpatrick skin type were collected. Wound mapping was performed with clinical photographs and the dimensions were recorded according to the surface area.

**Table 1.** Patient eligibility criteria

Patient inclusion criteria	Patient exclusion criteria
Age: 18–65 y Full-thickness wounds following burn wound/scar excision Deemed compliant with complex after-care Capacity to give informed consent for the research trial, photography, and skin biopsies	Allergy to bovine collagen Inhalation injury at the time of burn Deemed unlikely to survive study period Risk factors that can affect healing Auto-immune disorders or immunosuppressive states Long-term antiplatelet or anticoagulation therapy Inclusion in another research trial Pregnant or breastfeeding

### Procedure

**Figure 1.**
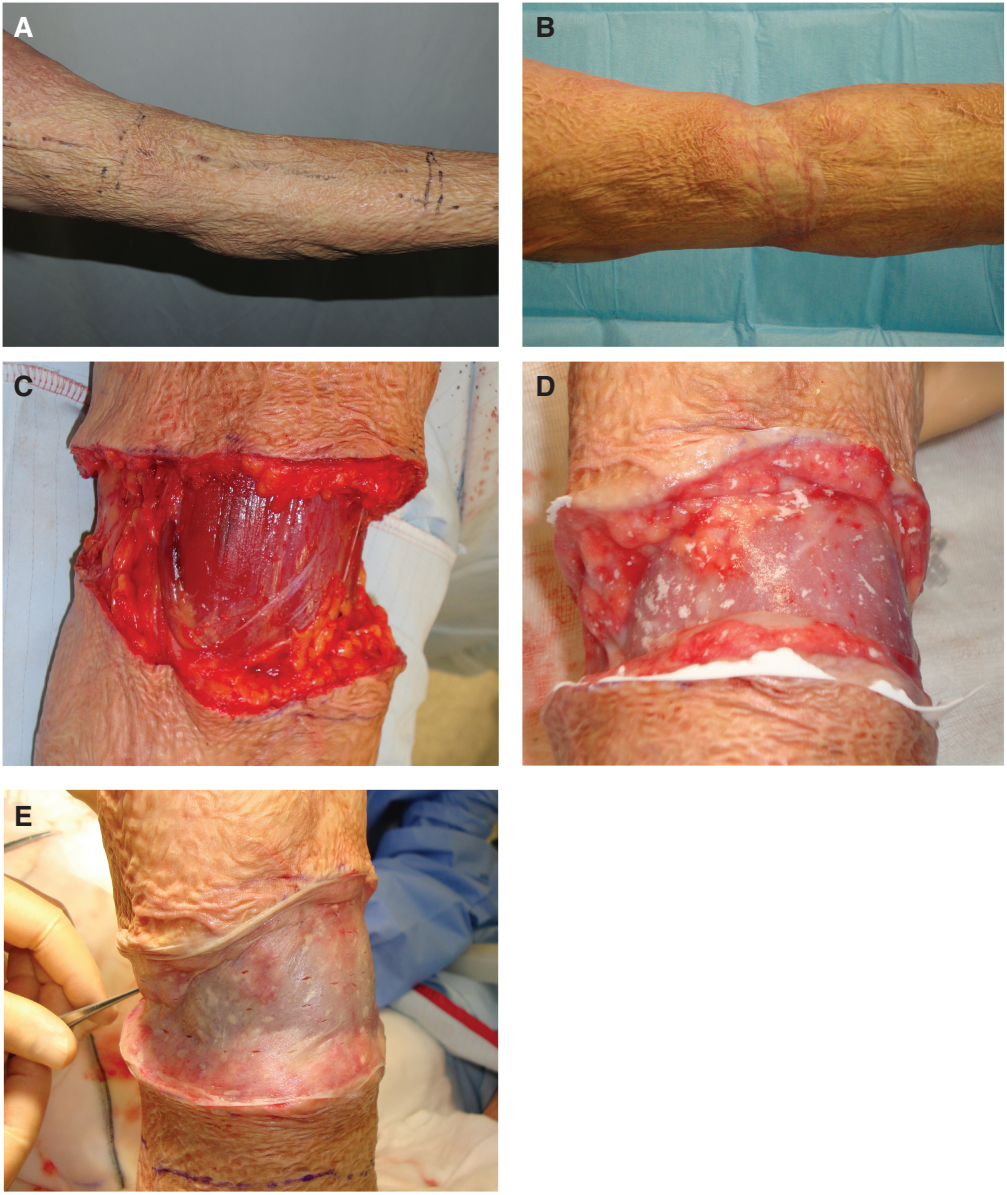
Surgical application of MatriDerm from a single patient (M05): A. Preoperative appearance with scar contracture; B. Wound bed preparation; C. MatriDerm® application; D. SSG application; and E. Month 3.

The surgical protocol is demonstrated in Figure 1A–D. Surgical excision of burn scar tissue (Figure 1A) was performed in depth to a healthy wound bed of either fat or in some cases fascia, and breadth to soft wound boundaries (Figure 1B).^34^ After meticulous haemostasis, Artiss® (Baxter Healthcare Ltd., Norfolk, UK) a fibrin sealant, was sprayed onto the wound bed to improve adherence and aid haemostasis. MatriDerm® of 1 mm thickness was then applied dry to the wound bed and moistened with saline in-situ to improve conformity (Figure 1C). MatriDerm® becomes impossible to handle if wetted before application and should only be moistened after it is placed well onto the wound. A second spray of Artiss® was then applied over the top of the matrix before the skin graft was applied. A sheet of epidermal autograft (0.06- to 0.08-inch thickness) was then applied (Figure 1D) and dressed with at least six layers of Bactigras® (Smith and Nephew plc, Watford, UK) and Polyfax® antimicrobial ointment (Elaiapharm, Valbonne, France). This particular primary dressing provides a sealed moist environment to prevent desiccation of the skin graft until the MatriDerm® is vascularized enough to support it. To prevent sheer and accumulation of fluid under the matrix or the skin graft, a foam cylinder or a tie-over dressing were applied according to the location of reconstruction. Patients were allowed home either on the same day or the day after for reconstructive cases.

Primary graft inspection was performed on day 7 postoperation, with utmost care to prevent sheer or displacement. Skin graft overlying the MatriDerm® matrix is extremely fragile. At this stage, the skin graft is usually adherent, if not, the graft was handled with extreme care and dabbed clean and the dressings replaced.

Patients were closely monitored by a senior occupational therapist, before, during, and after the procedure. Scar management commenced the moment the skin graft was stable and solidly adherent to the underlying neodermis (Figure 1E).

### Clinical Assessments

All patients were scheduled for 12 months of follow-up (weeks 1, 2, 3, and 4; months 2, 3, 6, 9, and 12). Clinical images were taken and a modified Vancouver Scar Scale (mVSS) was documented at each visit. Patient satisfaction in subjects undergoing scar reconstruction was assessed using a questionnaire.^33^ The mVSS and Patient Satisfaction Scores were documented by either a surgeon, senior research nurse, or occupational therapist experienced in scar management but not involved in the care of the study patients.

### Biopsies

At each visit, a single punch biopsy (3 mm) was performed under local anesthesia from the skin-grafted site, allowing at least a 1 cm border of MatriDerm® to be left around the edge of the site. The specimen was placed in 10% formalin saline solution and paraffin sections were processed with hematoxylin and eosin (H&E), Elastic-Van-Gieson (EVG), and Orcein staining. Immunohistochemical analysis was performed to determine neovascularization, using antibodies to the vascular endothelial cell marker CD31. MatriDerm® thickness, vessel size, and density were measured. The neodermis and MatriDerm® thickness were measured in the H&E, Orcein, or EVG stained slides (with an average of 9 thickness measurements per slide); whilst vessel size and vessel density measurements were obtained in the CD31 stained slides using Image J software. Additionally, representative slides were imaged with transmission electron microscope (TEM) and collagen fiber (native and MatriDerm®) thickness was measured. All measurements were performed by two independent assessors and the mean readings were used.

### Statistical Analysis

Longitudinal data were analyzed using the Statistical Package for Social Sciences Software version 19 (Armonk, NY: IBM Corp) using Wilcoxon signed rank tests and *T*-tests. A *P*-value of <0.05 was considered significant.

## RESULTS

Ten sites of MatriDerm® reconstruction were performed on eight patients. The patients were mostly male, with a mean age of 34.5 years (range 18–55 years) and no significant preexisting physical comorbidities. Fitzpatrick skin type and anatomical site of reconstruction varied across the cohort (Table 2). With the exception of one case for acute burn wound cover, all cases were performed for burn scar reconstruction.

**Table 2.** Patient baseline characteristics

Patient	Age	Gender	Comorbidities	Fitzpatrick Skin Type	Anatomical Site of Reconstruction	Type of Reconstruction
M1	33	Male	None	3	Right upper thigh	Acute burn
M2^*^	53	Male	None	4	Left arm	Scar reconstruction
M3	38	Male	Psychiatric disorder	3	Left popliteal fossa	Scar reconstruction
M4	38	Female	None	1	Left anterior and lateral thigh	Scar reconstruction
M5^#^	25	Male	None	3	Left axilla, radial upper arm, forearm	Scar reconstruction
M6	25	Female	None	4	Left anterior torso	Scar reconstruction
M7	30	Male	None	5	Left forearm	Scar reconstruction
M8^*^	55	Male	None	4	Right popliteal fossa	Scar reconstruction
M9^#^	26	Male	None	3	Right arm	Scar reconstruction
M10	18	Female	None	5	Right breast	Scar reconstruction

^*^M2 and M8 are the same patient operated on at different anatomical sites on different dates;

^#^M5 and M9 are the same patient operated on at different anatomical sites on different dates.

### Epidermis

The epidermis showed reconstitution of the rete ridges as early as 2 weeks, but the rete ridges seen at this stage were flattened and sparse. By week 4 all specimens showed relatively well-developed rete ridges.

### Dermis

**Figure 2.**
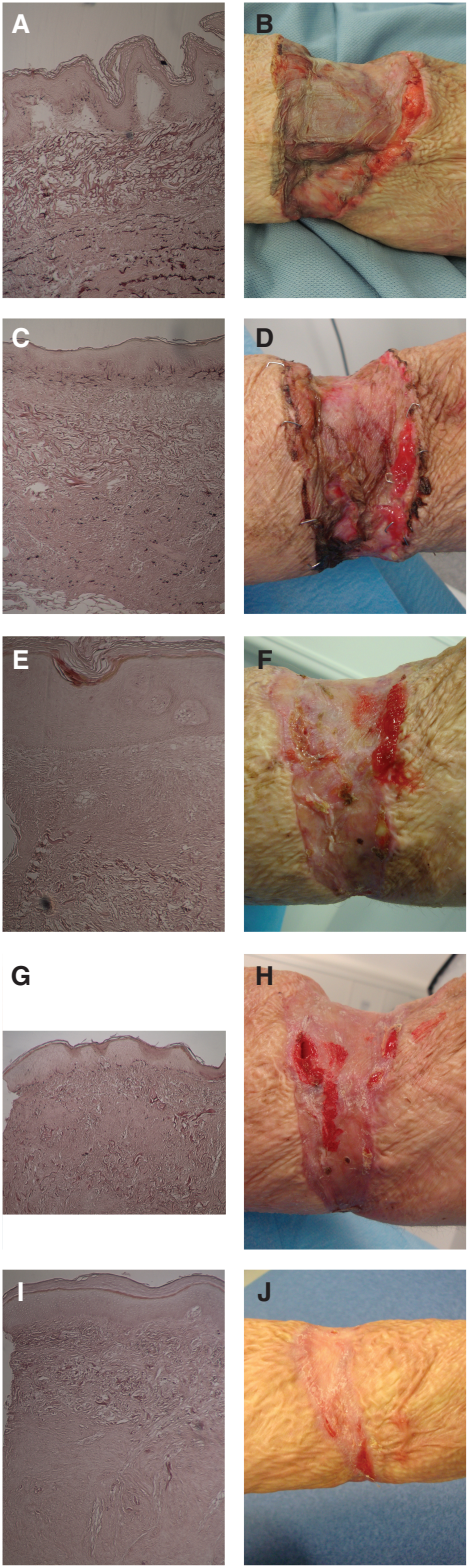
MatriDerm® histology at ×10 magnification using Orcein staining with accompanying clinical appearance from a single patient (M05): A. Week 1 histology; B. Week 1 clinical appearance; C. Week 2 histology; D. Week 2 clinical appearance; E. Week 3 histology; F. Week 3 clinical appearance; G. Week 4 histology; H. Week 4 clinical appearance; I. Month 2 histology; and J. Month 2 clinical appearance. MatriDerm® layer integrity is preserved up to week 2 and shows distortion and fragmentation from week 3 onwards. At month 2, MatriDerm is entirely replaced by neodermis.

**Figure 3.**
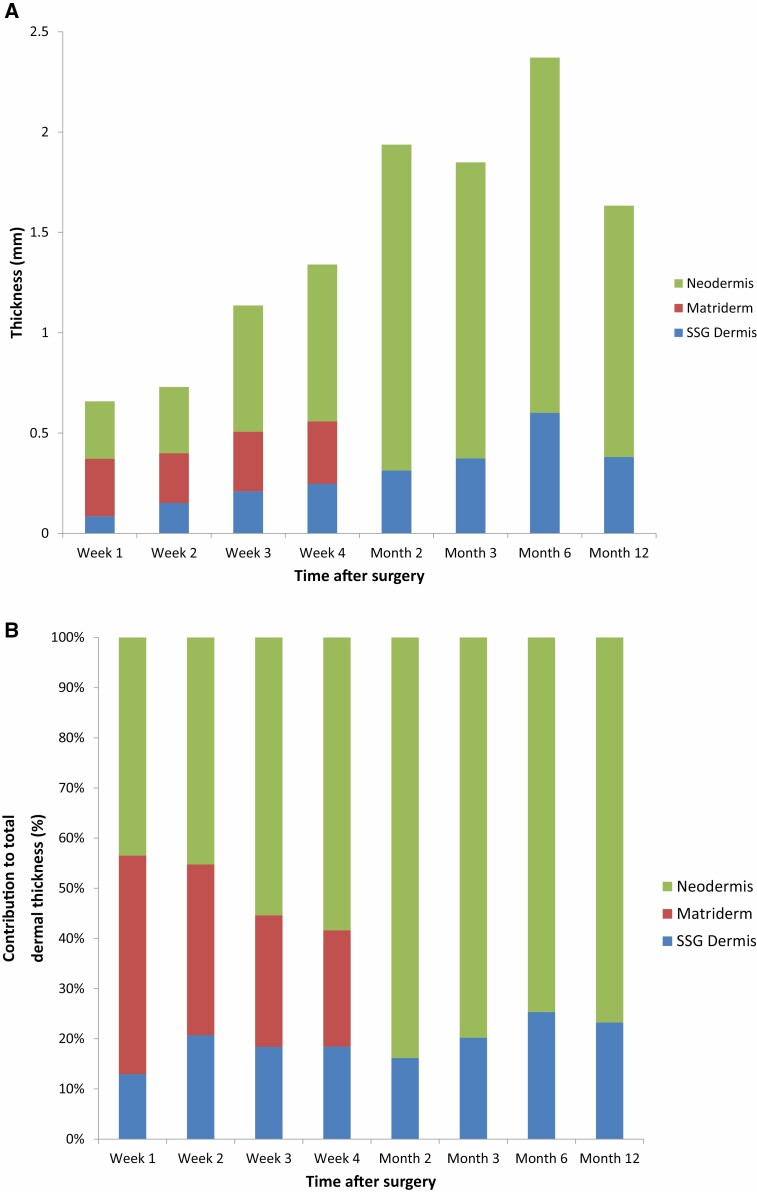
Total dermal thickness with varying contributions of the skin graft dermis, MatriDerm® and neodermis in: A. Actual thickness (mm); B. Percentage of total dermal thickness (%).

The gradual change in the histological and clinical appearance of the MatriDerm® reconstructed wound of one patient is shown in Figure 2A–J, demonstrating by month 2 MatriDerm® has been resorbed and replaced by a neodermis. A significant increase in the thickness of the dermis from the skin graft (SSG dermis) is seen between week 1 (average 0.03 mm) and month 12 (0.38 mm) (*P* < 0.05) (Figure 3A). However, the relative contribution of the SSG dermis to the total dermal thickness is small, 13% at week 1 and 23% at month 12 (Figure 3B). Mean MatriDerm® thickness is seen to slightly increase from 0.26 mm (week 1) to 0.30 mm (week 4, *P* = 0.427) (Figure 3A). Significant resorption of MatriDerm® and replacement by neodermis is observed around weeks 3 to 4, when it becomes difficult to identify histologically (Figure 2G). As the MatriDerm collagen disappears, it is replaced with host collagen forming the neodermis that increases thickness from month 2 onwards. The dermal layer changes from three distinct layers (SSG dermis, MatriDerm, neodermis) to two layers (Figure 3B). No residual MatriDerm® is seen in any of the specimens at 12 months (Figure 2I).

### Vascularization and Inflammvatory Cells

**Figure 4.**
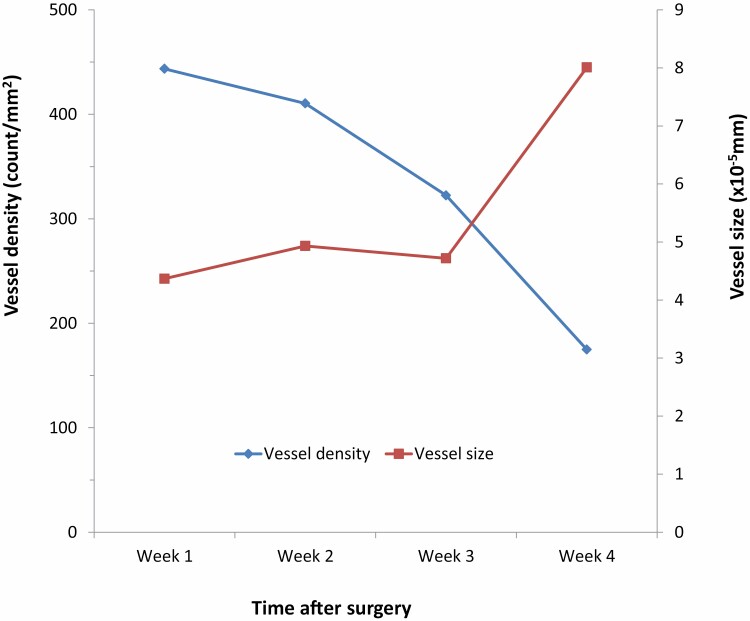
Mean vessel size and density over the initial four weeks after its application.

A high proportion of CD31 positive endothelial buds was observed in weeks 1 to 2 throughout the full thickness of MatriDerm®, with gradual vessel size increase (45.1 × 10^–5^ mm^2^, week 1 versus 79.3 × 10^–5^ mm^2^, week 4; *P* = 0.027) accompanied by no significant change in vessel density (529 vessels/mm^2^, week 1 vs 316 vessels/mm^2^, week 4; *P* = 0.161) (Figure 4). Inflammatory infiltrate of predominantly neutrophils with eosinophils, lymphocytes, and fibroblasts, was observed up to week 4, but seen only sporadically following this.

### Collagen and Elastin

All specimens showed increased collagen density in the lower reticular dermis. The collagen in the SSG dermis retains its random collagen arrangement at 12 months, however, the MatriDerm® layer and neodermis exhibit a nodular pattern of arrangement.

**Figure 5.**
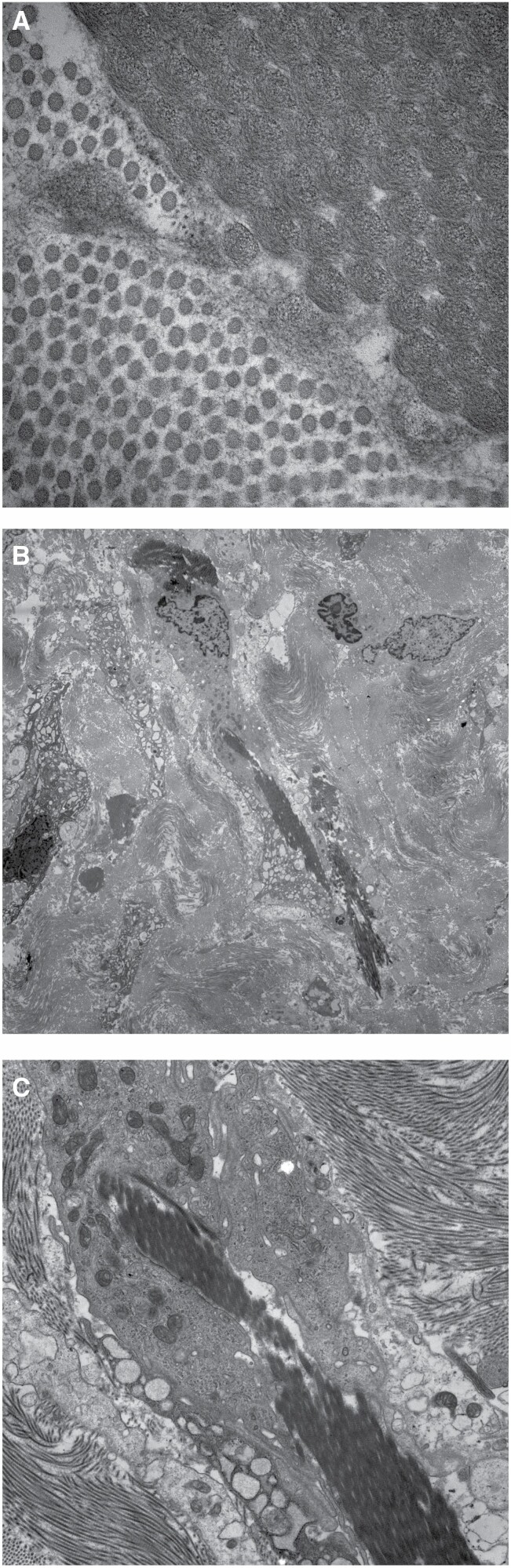
Transmission electron microscope images of MatriDerm® within the wound at direct magnification ×1900: A. MatriDerm® collagen bundles are larger in size compared to native collagen; B. Fragments of MatriDerm® are seen dispersed within the extracellular matrix during late stage of scar maturation; C. Macrophage observed engulfing bundles of MatriDerm® collagen.

With TEM, individual MatriDerm® collagen fibers were found to be significantly thicker than native collagen fibers (mean diameter: 172 nm vs 53 nm, *P* < 0.001) (Figure 5A). As the scar matures MatriDerm® collagen fibers are seen to have fragmented and dispersed (Figure 5B). Macrophages are seen phagocytosing MatriDerm® derived collagen (Figure 5C).

Elastin fibers were present in 78% of specimens (7/9), however, the fibers seen were short and thickened with reduced density in all specimens. Mature elastic fibers could not be identified with the TEM.

### Clinical Outcomes

There was no significant change in mean mVSS over the study period (preoperative, 7 ± 3 vs month 12, 7 ± 3; *P* = 0.310). The majority of patients reported improved outcomes following reconstruction with regards to their experience of itching, pain, appearance, dryness, pliability, sensation, and activities of daily living (Table 3). All patients who had difficulties in performing activities of daily living at screening reported improvement after surgery.

**Table 3.** Patient self-assessment survey of scar improvement; significance = *P* < 0.05

Parameter (Total *n*)	Improved (%)	No Change (%)	Worsened (%)	*P*
Itch (8)	62.5	25.0	12.5	0.62
Pain (9)	55.6	22.2	22.2	1.00
Appearance (9)	66.7	11.1	11.1	0.35
Dryness (9)	77.8	22.2	0.0	0.06
Softness (9)	55.6	33.3	11.1	1.00
Sensation (9)	66.7	66.7	0.0	0.35
ADL (9)	110	0	0.0	<0.01

## DISCUSSION

The invention of the first dermal substitute, Integra® in the 1960s was a remarkable step change in wound management of acute major burns, reconstructive surgery, trauma, and chronic wounds. Improvements in surgical techniques have widened the scope of its application and avoided otherwise complex procedures in frail patients. Clear histologic understanding of how dermal substitutes are integrated onto the wound bed should improve day to day management of our patients and gives an insight of future dermal substitute developments. This study was designed to mirror the methodology of previously published work investigating the histological integration of Integra® into the wound bed.^3^ One-millimeter thick MatriDerm® was applied to nine postburn reconstruction sites, and one case of acute burn wound coverage. Sequential histological examination to evaluate the integration of MatriDerm® was performed. This was correlated with the Integra® previous histologic study (Table 4). At the time of study design single layer Integra® had not yet received a “Conformite Europeenne” certification and was therefore not available for inclusion. A comparative study may give an insight into the different vascularization patterns of the two matrices, which might help future dermal substitute characterization.

**Table 4.** Phases of integration of dermal regeneration templates MatiDerm® and Integra® within the reconstructed burn scar

Timeline	MatriDerm®	Integra®
Week 1	Imbibition Inflammatory infiltrate of predominantly neutrophils. MatriDerm® remains of equal thickness in the wound	Imbibition Matrix interstices fill with wound fluid and matrix swells Fibrin fosters adherence of matrix to wound
Week 2	Lymphocytes, macrophages, occasional eosinophil, and fibroblasts are seen Rete ridges appear CD31 seen with patent vascular channels that traverse the MatriDerm® matrix	Fibroblasts seen in the wound using Integra® collagen as a scaffold Endothelial cell migration seen from the end of the second week
Week 3	Beginning of resorption and replacement by neodermis A sharp increase in vessel size with no increase in vessel density	Fibroblasts seen settled along the interstices of the matrix and producing host collagen
Week 4	Immature collagen bundles seen	Solid columns form that stain positive for CD31 Lumen formation is seen by the fourth week New vessel formation is occasionally seen by the end of the fourth week
Week 4 onwards	MatriDerm® continues to resorb and by 2 months is completely by neodermis	New collagen indistinguishable from the normal dermis Initially, neodermis is thicker than native dermis but thins over time Autograft becomes adherent to neodermis Rete ridges form

Histological analysis of this study demonstrated similarities between MatriDerm® vascularization, and Integra® (Table 4).^33^ The cellular infiltration and vascularization of the MatriDerm® matrix seemed to be accelerated, however the matrix loses its thickness soon after it is applied to the wound bed, a feature that was not seen when examining Integra® matrix histologically. Vascularization followed the same pattern in both matrices with increased vascular lumen size and reduced vessel density as the neodermis matures.

By the end of the second week, fibroblasts begin to infiltrate the MatriDerm® matrix, together with some inflammatory cells, predominantly neutrophils. Macrophages were noted in the third week as new collagen was laid down by invading fibroblasts and the matrix collagen was resorbed. There was early evidence of angiogenic activity with a high density of patent endothelial channels traversing the entire MatriDerm® scaffold, identified with CD31 cell markers. By the third week, during MatriDerm® resorption, there was a significant increase in vessel size, with no increase in vessel density. Similarly, endothelial cells identified with CD31 were present in Integra® at the beginning of the second week, with lumen formation seen during the third week. When examining Integra®, neovascularization was only well established at the end of the fourth week, corresponding with increased graft take when skin grafting was delayed to the fourth week.^33^

A major difference observed was the early resorption of the MatriDerm®, starting by the fourth week and almost complete at 2 months. Integra® matrix traces were still histologically present at 2 years postsurgery.^35^ This could be explained by the cross-linkage of the Integra® matrix that maintains its structure, avoiding collapse of the scaffolding whilst cellular infiltration into its porous structure is progressing. MatriDerm® collagen matrix, on the other hand, is not cross-linked, a design to promote faster cell infiltration into the matrix. The lack of cross-linkage may also explain the early reduction of the matrix thickness seen at weeks 1 and 2, and the early resorption of the collagen by month 2. The inflammatory cellular response demonstrated in this study was not seen in the previous Integra® histological study and previous animal study.^3,36^ This could be explained by the need for inflammatory cell infiltration to clear the debris of the collapsing scaffold and prepare the wound for the deposition of new collagen by the native fibroblasts. Alternatively, the addition of Glycosaminoglycan GAGs to Integra® collagen matrix may explain the lack of the inflammatory response and the resistance to early resorption.

The histological findings of this study support the use of 1 mm thick MatriDerm® as a single-stage procedure. The senior author’s experience with 2 mm MatriDerm® with immediate application of skin graft was disappointing, with significant skin graft loss. The 2 mm thick matrix is too wide a barrier to the imbibition required to maintain skin graft viability. This can be mitigated by applying an occlusive dressing to prevent skin graft desiccation till vascularization is enough to maintain skin graft viability. The presence of wide pores on the MatriDerm® matrix and its ability to hold wound exudate enhances early graft nutrition and survival prior to early vascularization, allowing one-stage reconstruction.

In this study, with the use of electron microscopy, elastin fibers seen in MatriDerm® were different to normal elastic fiber architecture, and were short, thickened, and reduced in density in all specimens. A recent study compared elastin fiber density in biopsies taken from patients who had reconstruction with MatriDerm®, full-thickness skin graft (FTG), SSG, Alloderm®, and normal skin. Elastin contents in MatriDerm® samples were almost half those of normal skin or FTG but more than SSG or Alloderm®.^37^ These results and the histological findings in our current study, question the effectiveness of the elastic fibers in the MatriDerm® matrix.

Only one patient experienced worsening of their symptoms of itch, pain, appearance, and softness. Without a comparison of scar reconstructed with SSG only it is difficult to conclude whether the patient-reported improvement is due to MatriDerm® or the nature of scar release. We found that reconstruction of a chronic burn scar with MatriDerm® did not alter mVSS at 12 months, in keeping with literature. In an intraindividual comparison of MatriDerm® reconstruction to standard skin grafting reconstruction, there is a documented early improvement in VSS at 3 to 4 months after reconstruction.^38,39^ However, this is not seen at 12 months, in keeping with our study.^40^

### Study Limitations

There are a number of limitations to this study. Histological analysis of revascularisation is a two-dimensional assessment of a complex three-dimensional process, and provides limited data in comparison to other techniques such as corrosion casting and the use of high-resolution episcopic microscopy.^7,41,42^ We did not use scar assessment tools such as the Cutometer® (Courage + Khazaka, Germany) which give an objective assessment of the clinical outcome of MatriDerm®.^43,44^ Instead, we used patient-reported outcomes and the Vancouver Scar Scale (VSS), the same clinical assessment methodology as for the previous study of Integra®.^3^ Scar assessment is complex, with currently no defined validated “gold standard”.^45^ Outcomes that are important to patients encompass more than scar characteristics, making patient-reported outcomes the most clinically relevant.^46^ The mVSS, despite questionable reliability, is a straightforward and low-cost tool that is widely used, making its inclusion useful for comparison amongst the literature.

### Conclusion

This study demonstrates the stages through which MatriDerm® integrates within the reconstructed burn scar. The early angiogenic activity observed in this study gives evidence to support the one-stage reconstruction model of MatriDerm®. It was found to have resorbed from the tissue bed and was absent from the reconstructed site completely by the end of the second month. Future biomechanical and immunological studies can investigate the mechanism of neo-dermal synthesis and help characterize the ideal dermal substitute matrix.
